# Analysis of Primary Stability in Two Designs of Ultrashort Implants: In Vitro Study

**DOI:** 10.3390/bioengineering13060606

**Published:** 2026-05-23

**Authors:** Paula López-Jarana, Rui Pedro Marques, Reyes Jaramillo, Rocio Santos, Juna Barros, André Matos, Daniel Robles-Cantero, Mariano Herrero-Climent

**Affiliations:** 1 Master 360 Symmetrya-Porto, Faculty of Health Sciences, Miguel de Cervantes European University, 47012 Valladolid, Spain; plopezjarana@hotmail.com (P.L.-J.); ruipedromarques.dds@gmail.com (R.P.M.); junacatsilbarros@gmail.com (J.B.); andresilvamatoss@gmail.com (A.M.); mariano.herrero@herrerocliment.com (M.H.-C.); 2 Department of Periodontology, Universidad de Sevilla, 41009 Seville, Spain; mrjaramillo@us.es (R.J.); rsantos3@us.es (R.S.); 3 DENS-iA Research Group, Faculty of Health Sciences, Miguel de Cervantes European University, 47012 Valladolid, Spain

**Keywords:** ultrashort implants, primary stability, insertion torque, Implant Stability Quotient, drilling protocol, bone density

## Abstract

Background: This in vitro study evaluated the influence of macro and microscopic implant design, drilling protocol, and bone density on the primary stability of 4 mm ultrashort dental implants, aiming to provide evidence-based guidance for their use in severely atrophic posterior jaws. Methods: Two implant systems were compared: test group (Klockner Essential Cone^®^ conical implants with polished neck, diameters 4.0 mm [B1] and 4.5 mm [B2], shot-blasted and acid-passivated surface) and control group (Straumann Standard Plus^®^ 4.1 mm parallel-walled implants with SLA^®^ surface). A total of 722 implants (*n* = 30 per condition) were inserted into natural bone blocks simulating Lekholm and Zarb type II (cortical-dominant) and type III (medullary-dominant) bone qualities. Fifteen experimental conditions were tested, combining three main drilling protocols: (1) manufacturer’s standard preparation, (2) horizontal under drilling (final diameter 3.5 mm), (3) vertical overpreparation (1 mm deeper), and (4) combined vertical + horizontal restriction. Primary stability was assessed by insertion torque (measured with a calibrated Tohnichi^®^ torque wrench) and Implant Stability Quotient (ISQ) using Penguin^®^ resonance frequency analysis (RFA) in two perpendicular directions. Subjective insertion ease and complications were also recorded. Conclusions: The conical macrogeometry with progressive, dense V-shaped threads provides significantly better primary mechanical anchorage than parallel-walled designs in ultrashort (4 mm) implants. Within the limitations of this ex vivo animal bone model study, the results indicate that different drilling protocols significantly influence the primary mechanical stability with insertion torque ≥ 25 Ncm and ISQ ≥ 55 of ultra-short implants, as measured by insertion torque and ISQ values. Certain drilling protocols resulted in higher insertion torque and ISQ compared to others, particularly in Type II and Type III bone.

## 1. Introduction

When bone atrophy is severe in the posterior regions of the jaws, the use of even short implants is limited, generating the need to use devices even smaller, commonly called ultra-short implants (≤6 mm) [1,2,3,4,5]. Several studies have evaluated their clinical behavior and survival rates, reporting encouraging results. A recent systematic review indicated a survival rate average of 94.1% at five years, with an average marginal bone loss of 0.53 mm [6]. Concordantly, a five-year retrospective follow-up study compared 5 mm and 6 mm long implants in posterior areas, showing survival rates of 95.6% and 95.65%, respectively [7,8,9,10].

Despite its advantages—less surgical invasiveness, less morbidity, and reduction in treatment costs—ultra-short implants can be associated with specific complications, such as implant loss and marginal bone resorption.

Factors such as length, diameter, and crown-implant ratio (C/I) significantly influence their clinical behavior [11,12,13]. In addition, the limited bone-contact surface and the high C/I ratio in implants ≤ 6 mm continue to generate uncertainty about their long-term survival. Therefore, although ultra-short implants constitute a viable therapeutic alternative in cases of severe atrophy, their indication must be carried out under careful case selection and respecting the established biomechanical and surgical principles [14,15]. Although ultra-short implants constitute a viable therapeutic alternative in cases of severe atrophy, their limited bone contact surface and high crown-to-implant ratio (C/I) continue to generate uncertainty about their long-term survival [14,15]. Recent combined in vitro and finite element studies using the same Klockner Essential Cone^®^ 4 mm implants have confirmed that C/I ratios of 4.55 significantly increase micromotion and peri-implant bone micro-strain compared to longer implants, although prosthetic splinting reduces both parameters to levels comparable to 6–10 mm implants [16]. To optimize this integration, various strategies have been developed based on modifying the macro- and micro-design of titanium implants [17]. The macro-design includes characteristics such as length, diameter, morphology, internal angle, thread height and pitch, groove depth, helix angle, and connection type. These parameters have a direct influence on primary stability by determining the shape and extent of contact between the implant and the recipient bone, promoting efficient mechanical fixation and proper distribution of occlusal loads [18,19]. The evidence consistently demonstrates that bone quality is one of the two main determinants of primary stability of short dental implants. Most of the studies analyzed evidence a direct relationship between bone density and parameters such as insertion torque and ISQ, confirming that higher-density bones are associated with better initial anchoring conditions.

Studies such as those by Anitua et al. [20] and Alonso et al. [21] will show that higher values of torque and ISQ are only obtained in types I and II, while those with lower density, namely types III and IV, present lower levels of initial stability. These results are consistent with the expected biomechanical behavior of bone tissue, since lower density implies lower resistance to implant insertion and lower initial contact between implant and bone.

The use of extra-short implants, particularly those of 4 mm, continues to generate controversy regarding their clinical predictability. Their reduced bone contact surface and high crown-to-implant ratio make them especially dependent on adequate primary stability [11,22]. However, most available studies focus on short implants between 6 and 8 mm, with little specific scientific evidence on 4 mm implants [14,23]. This lack of evidence is especially relevant to the influence of the macro design and of the drilling techniques on primary stability. Furthermore, existing research often presents heterogeneous methodologies and multiple uncontrolled variables, hindering direct comparison between different approaches and limiting the practical applicability of the results [18].

This in vitro study proposes a systematic and reproducible experimental approach aimed at isolating critical variables—such as implant design, drilling protocol, and bone density—to ensure high internal validity. In this way, it seeks to fill a significant gap in the literature and provide solid evidence to support informed clinical decisions, thus contributing to improved predictability and long-term success of oral rehabilitation in cases of severe bone atrophy.

The main objective is to systematically investigate the use of 4 mm extra-short implants, evaluating how macroscopic and microscopic design influences primary stability and the predictability of their survival in type II and III bone conditions, through an in vitro experimental study. The specific objectives were: (I) to compare the primary stability of implants with different designs and diameters, (II) to measure insertion torque and ISQ as stability indicators, (III) to evaluate the impact of different drilling protocols (standard, vertical and horizontal undercut) on implant stability and ease of insertion, and (IV) to determine the mechanical behavior in different bone densities (type II and type III bone), simulating clinical situations of atrophic maxillary and mandibular ridges. The null hypothesis (H_0_) of the present study is that there are no statistically significant differences in the primary stability—as measured by insertion torque and Implant Stability Quotient (ISQ) values—of ultra-short implants with different macro- and micro-designs, drilling protocols, and bone densities (Type II and Type III). The alternative hypothesis (H_1_) is that there are statistically significant differences in primary stability depending on implant designs, drilling protocols, and bone density.

## 2. Materials and Methods

The study design is an in vitro experimental study that evaluates how the macro and microscopic design of 4 mm extra-short implants influences primary stability and clinical predictability.

### 2.1. Bone Model

To simulate atrophic maxillary/mandibular bone, ribs and hip bones were used at two different densities, according to the Lekholm and Zarb classification, designed to describe bone quality. The substrates were: (a) type II bone, characterized by intermediate density where the cortical component predominates, and (b) type III bone, with a greater medullary component and therefore reduced bone density. A key advantage of using homogeneous bone is that it ensures high reproducibility and minimizes the variability inherent in using biological specimens. The short dental implants were inserted into fresh bovine ribs simulating bone quality type II and fresh femoral epiphysis simulating bone quality type III (by Leckholm and Zarb). Bone quality was clinically assessed according to the Lekholm and Zarb classification during osteotomy preparation. The type II bone presented a thick cortical layer surrounding dense trabecular bone with moderate to high resistance. Type III bone showed a thin cortical plate and medium-density trabecular bone with moderate resistance to drilling. This clinical classification was subsequently confirmed by the insertion torque and initial ISQ values recorded at implant placement. The simulation was performed in a room with a controlled ambient temperature (23 °C) to simulate body temperature in the bone substrate.

### 2.2. Implant Groups Studied

The behavior of two groups of extra-short implants, 4 mm in length, was analyzed. One test group consisted of Klockner Essential Cone (EC)^®^ implants (SOADCO S.L., Andorra) with polished necks in two diameters: 4.0 mm and 4.5 mm (B1 and B2). Additionally, a control group was included with Straumann Standard Plus (SP)^®^ implants (Straumann Group, Basel, Switzerland), 4.1 mm in diameter. Klockner EC implants are macroscopically characterized by their conical design, with a V-shaped threaded body and a 2.2 mm machined transmucosal neck. Microscopically, they feature a titanium surface treated with shot blasting and acid passivation. Control group implants have a straight body with double-helix threads and a smooth neck with a height of 1.8 mm; they are made of pure titanium with an SLA^®^ surface. The Straumann ultra-short implant was selected as the control group because it represents one of the most widely studied and clinically validated ultra-short implant systems currently available on the market. In a previous pilot study, the minimum sample size (*n* = 27) was determined for statistical significance (*p* = 0.05), and a confidence interval was set at 95% by means of the software package N-Query Advisor 6.0. Sample size was determined by means of Bonett’s formula (10), and data analysis was carried out with the software package SPSS 17.0 for MS Windows (SPSS, Chicago, USA). Twelve different experimental conditions were analyzed with a sample size of *n* = 722 implants (30 implants per group) to ensure adequate statistical power [24,25].

### 2.3. Surgical Protocol

Implant preparation was performed using original Klockner^®^ and Straumann^®^ burrs, calibrated with depth stops up to 4 or 5 mm to standardize the preparations. A PCP 15 mm probe was used to control the preparation depth.

Bone blocks were prepared and fixed in a stable device. Drilling of the implant site for all experimental groups (L1–L12) was performed according to the manufacturer’s protocol. Two variables related to primary stability were determined. A Tohnichi^®^ ATG6C manual torque wrench was used to determine the insertion torque, and a Swedish-made Penguin^®^ RFA (Integration Diagnostics Sweden AB, Gothenburg, Sweden) was used to determine the stability (ISQ) of the dental implants. The ISQ assessment was determined in two perpendicular directions on the multipeg, taking the average of the values.

**Figure 1:**
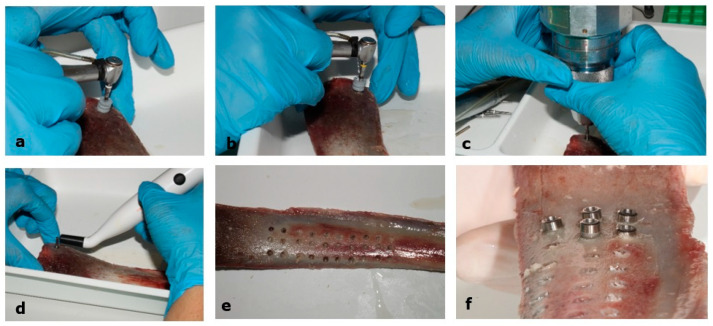
Implant bed preparation: (**a**) pilot drill and depth stop, (**b**) successive drill with stop, (**c**) measurement with torque wrench, (**d**) ISQ measurement with Penguin, (**e**) 30 perforations made per group, and (**f**) implants inserted.

Multipegs were used for Klockner (ref. 55067-59) and Straumann (ref. 55014-4) implants. A qualitative record of clinical perception was also made, including ease of insertion, implant rotation, and variations in depth. Photographic documentation was taken of all critical phases (Figure 1).

### 2.4. Experimental Groups

The implants studied are presented, as well as the different drilling protocols: standard, horizontal, and vertical subpreparation, twelve groups for bon type II and twelve groups for bone type III [24] (Table 1).

### 2.5. Study Variables

Both dependent and independent variables were considered in the study. Dependent variables included insertion torque (TI), resonant frequency analysis (ISQ), operative complications, and subjective stability as perceived by the operator. Independent variables included macroscopic and microscopic implant design, bone density (type II vs. type III), and drilling protocol (standard; 5 mm vertical over-preparation; 3.3–3.5 mm horizontal under-preparation; and 5 mm vertical over-preparation + 3.3–3.5 mm horizontal under-preparation). Insertion torque (TI) is a primary dependent variable representing bone resistance to implant advancement. ISQ was measured in two perpendicular directions to improve reproducibility.

### 2.6. Statistical Analysis

In this study, the torque and average ISQ of the implants were evaluated. Initially, the Shapiro–Wilk test was applied to determine the normality of the data for both variables, which allowed us to decide whether to proceed with parametric or nonparametric tests. Regardless of the normality result, the Kruskal–Wallis test was used as the primary method to compare the medians between the study groups, with the aim of identifying statistically significant differences in these variables. Additionally, an analysis of variance (ANOVA) followed by Tukey’s post hoc test was performed, which confirmed the presence of significant differences between the analyzed groups. This combined approach ensures a robust evaluation in both normally and non-normally distributed data scenarios. Data normality was assessed using the Shapiro–Wilk test and quantile-quantile plots. In Type III bone, both insertion torque and mean ISQ values violated the normality assumption (Torque: W = 0.84215, *p* < 0.001; ISQ: W = 0.67257, *p* < 0.001). Therefore, non-parametric tests were applied. The Kruskal–Wallis test was used to evaluate differences among drilling protocol groups, followed by Dunn’s post hoc test for pairwise comparisons. In Type II bone, insertion torque values met the normality assumption (W = 0.98303, *p* = 0.136), whereas mean ISQ values did not (W = 0.69572, *p* < 0.001). For consistency across the study, both parametric (one-way ANOVA with Tukey post hoc) and non-parametric (Kruskal–Wallis with Dunn’s post hoc) tests were performed for insertion torque. For ISQ values, only non-parametric tests (Kruskal–Wallis followed by Dunn’s test) were used. All analyses were conducted using SPSS software version 26.0, with the significance level set at *p* < 0.05.

## 3. Results

The statistical analysis aimed to evaluate the effects of different drilling protocols on insertion torque and primary stability (average ISQ) of different types of short implants placed in type II and III bone.

### 3.1. Effect of Drilling Protocol by Implant Type and Bone Quality

**Figure 2:**
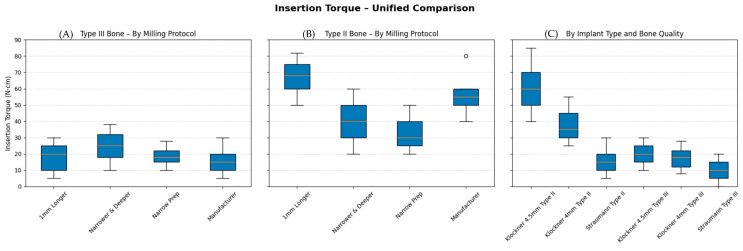
Insertion torque: (**A**) Type III Bone-By drilling protocol. (**B**) Type II Bone-By drilling protocol. (**C**) By implant type and bone quality.

In type III bone, torque showed significant differences between protocols in all groups (*p* < 0.05 in test and control), with higher values in “narrower and deeper” and “1 mm longer” preparations vs. the manufacturer’s protocol (Figure 2A). The ISQ presented greater variability, but only significant differences in test implants (*p* < 0.001), with superior stability in more restrictive drillings. In type II bone, the torque was consistently higher with undermilled length/depth (*p* < 0.001 in the majority), although the ISQ showed significant differences mainly in test implants (Figure 2B).

### 3.2. Comparison Between Implants and Bone Qualities

Under the manufacturer’s protocol, the test implants (conical design) presented significantly greater torque than the control (parallel walls) (*p* < 0.001), especially in type II (median 45 Ncm vs. 20 Ncm in 4.5 mm test vs. control). The ISQ followed a similar trend but with greater dispersion in the control (Figure 2C). Both the type of implant and bone quality significantly influenced torque and ISQ (*p* < 0.001), with an interaction between both factors. Torque and ISQ decreased notably in type III, but test implants maintained higher values. In Type III bone, Kruskal–Wallis tests demonstrated significant differences among drilling protocols for insertion torque (H = 9.08, *p* = 0.028; Table 2) and mean ISQ (H = 30.77, *p* < 0.001; Table 3). In Type II bone (B2), significant differences were found for both insertion torque (H = 71.75, *p* < 0.001; Table 4) and mean ISQ (H = 24.19, *p* < 0.001; Table 5). In Type II bone (B1), significant differences were also observed in insertion torque (H = 43.58, *p* < 0.001; Table 6) and mean ISQ (H = 9.05, *p* = 0.029; Table 7).

### 3.3. Global Effect or the Type of Drilling

Controlled underdrilling (deep stretching) significantly increased torque and ISQ in both bone types (*p* < 0.001), with a greater effect on test implants and in type II implants. No signs of excessive overcompression were observed.

**Figure 3:**
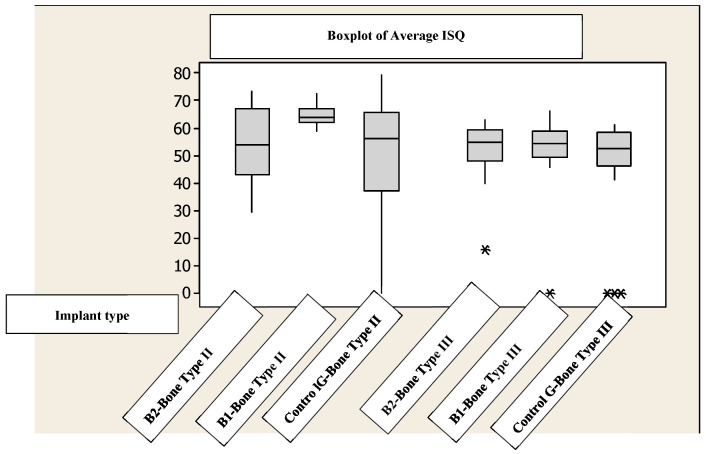
Boxplot graph of the average ISQ variable for the different types of implants and bone qualities under study.

Figure 3 presents the distribution of the implant stability index (average ISQ) according to implant type and bone quality (type II and type III). In cases corresponding to type II bone, ISQ values were generally higher, with medians ranging from 55 to 70. The Test B1 implant (4 mm) presented the highest median and lowest dispersion, indicating high and homogeneous primary stability. The Control group implant showed a wider dispersion, with values ranging from 30 to 80, reflecting greater variability among the samples. The Test B2 implant (4.5 mm) achieved an intermediate median (55) with a moderately wide distribution. In cases of type III bone, a general decrease in average ISQ was observed across all implant types, with medians close to 45–55. This behavior is consistent with the lower density and mechanical quality of this bone type. Furthermore, low outliers were identified, especially in the Control group, indicating cases with poor initial stability. The Test B2 and Test B1 implants showed similar results, with moderate variability and no marked differences between them.

## 4. Discussion

The need to rehabilitate atrophic posterior maxillae with less invasive treatments has led to the use of extra-short implants. This in vitro study analyzed the mechanical behavior and primary stability of 4 mm extra-short implants in bone with different densities (grades II and III) and under different drilling protocols. The results obtained allow for the analysis of the impact of the implant’s macro- and microscopic design, as well as the implant site preparation, on insertion torque (TI) and primary stability (ISQ), providing valuable information for the clinical management of severely atrophic ridges [25].

According to the results obtained in this study, the drilling protocol significantly influences the implant’s insertion torque, while its effect on primary stability, measured by ISQ, is more limited and depends more on bone density and the implant’s macroscopic design [13,24,26,27]. The highest torque values were observed in the “1 mm longer” and “narrower and deeper” drilling protocols, especially in type II bone, where the differences were statistically significant (*p* < 0.05). These results are consistent with those described by other authors, where a reduction in the prepared bed diameter increases bone-implant friction, improving initial mechanical stability [28,29]. However, there is a greater risk of bone crest compression, which can result in necrosis [30,31]. In the present study, thermal control, irrigation, and standardization of the drilling depth minimized these risks, supporting the clinical applicability of the proposed protocol.

In this study, the insertion of 4.5 mm prototype B2 implants using horizontal undercutting protocols in type II bone presented notable clinical difficulties. The absence of profiled burs in all groups necessitated the application of greater torque to achieve complete implant placement, generating resistance and a risk of bone overcompression in some cases. These findings are consistent with previous studies that have reported an increase in insertion torque in under-milled preparations without a specific profile, especially in intermediate-density bone (type II), which can hinder clinical manipulation and increase the risk of cortical microfractures [32]. Furthermore, it has been observed that this increased torque does not always translate into greater primary stability as measured by ISQ, demonstrating that the stress distribution around the implant depends on both bone density and the implant’s macro design [33,34]. Therefore, although horizontal undercutting can increase initial mechanical fixation, its use requires caution in extra-short implants with larger diameters to avoid complications related to excessive cortical bone compression.

Bone density played a determining role; torque was significantly higher in type II bone than in type III bone. This confirms the relationship between bone density and initial implant stability, already documented by other authors [25,31,35]. In the type III bone models, despite variations in the drilling protocol, ISQ values did not increase in this low-density bone, suggesting that initial stability depends more on the implant design than on the drilling protocol.

The use of the Penguin^®^ system allowed ISQ to be recorded in two perpendicular planes, increasing the reliability of the result, as recommended by recent studies on the reproducibility of resonant frequency devices. On the other hand, manual torque measurement with a Tohnichi^®^ torque wrench offered accuracy in the quantification of mechanical resistance, a method validated for comparative studies of primary stability [27,33,36].

When comparing the two implant systems studied, the test B2 implants exhibited the highest torque values, followed by the test B1 mm and, finally, the control group implants. This marked difference in insertion torque is predominantly driven by the macroscopic morphology of the protype B1 and B2 implant, which features a tapered/conical body with progressive threads, V-shaped thread profile, double spiral design, narrower thread pitch, and a higher number of threads (more aggressive and dense threading pattern) compared to the control implants, which exhibit a more parallel-walled (cylindrical) body, fewer threads, and a wider thread pitch. The conical progressive macrogeometry of the prototype promotes greater cortical bone compression and increased bone-implant friction during insertion, leading to significantly higher torque values and enhanced initial mechanical fixation, particularly in intermediate- and low-density bone [18,37]. In contrast, the control type design’s parallel walls and less dense threading result in lower frictional resistance and torque, reflecting a more uniform but less compressive stress distribution.

However, the correlation between torque and ISQ was not direct: in some cases, implants with high torque (especially the prototype) did not show proportional increases in ISQ. This can be attributed to the distinct stress distribution patterns induced by their microgeometries—the conical shape with denser, more aggressive threads in the test group concentrates compression at cervical and apical levels, while the parallel-walled morphology with fewer threads in the control group distributes stresses more evenly but with reduced initial frictional resistance. Torque primarily reflects insertion-related frictional and compressive forces (heavily modulated by the macroscopic conical design and thread density/pitch differences), whereas ISQ is more influenced by the overall stiffness of the bone-implant interface and cervical fit [5,36].

From a clinical perspective, modifying the manufacturer’s recommended drilling protocol by using narrower or deeper preparations can further enhance mechanical stability in low-density bone, particularly for implants in both test groups, where the conical macrogeometry, narrower thread pitch, and higher thread density maximize controlled compression and friction. Nonetheless, strict control of bone overheating and excessive compression remains essential to prevent necrosis, as the more aggressive conical threading of the prototype generates greater cortical forces than the parallel-walled control group design [9,12]. The present ex vivo study indicates that different drilling protocols can affect the primary stability of ultra-short implants, as measured by insertion torque and ISQ values. Nevertheless, because this is an animal bone model, these results cannot be directly extrapolated to clinical outcomes such as osseointegration or long-term implant success. The clinical applicability of these findings remains to be confirmed in future prospective human studies. The test implants (conical macrogeometry) consistently achieved significantly higher insertion torque and ISQ values than the control group. These results align with those reported by a recent in vitro study [16], which, using identical Klockner Essential Cone^®^ 4 mm implants in bovine type II bone, obtained a mean ISQ of 62 ± 4.4. Although lower than longer implants (ISQ 68.4 for 10 mm), this value remains above the clinical threshold of 60 considered acceptable for conventional loading [16].

It is important to note that, as this is an in vitro study, it has several limitations when interpreting the results. Therefore, the torque and ISQ values should be considered with some caution, as biological factors such as bone remodeling or functional loading are not considered. Nevertheless, it provides experimental evidence on how variations in the drilling protocol and implant design influence the primary stability of extra-short implants, improving surgical protocols in clinical situations of severe bone atrophy. Although the present study provides relevant information regarding the influence of drilling protocols on the primary stability of ultra-short implants, several limitations must be acknowledged. First, as an ex vivo animal bone model, this study evaluated only mechanical primary stability and did not assess biological responses such as bone remodeling, vascularization, or osseointegration over time. Second, differences between the two implant systems may exist beyond macrogeometry, including variations in microtopography, surface treatment, and thread design, which were not fully evaluated in this study. Third, although insertion torque was measured with a torquimeter, the possibility of subjective bias during manual implant insertion cannot be completely ruled out. Finally, because this was an in vitro study using animal bone blocks, the results should be interpreted with caution and cannot be directly extrapolated to clinical scenarios in humans, where factors such as bone quality heterogeneity, soft tissue response, and long-term healing play a critical role. Future in vivo and clinical studies are necessary to confirm the clinical relevance of these findings [38]. The results of the present ex vivo study suggest that different drilling protocols can significantly influence the primary mechanical stability of ultra-short implants, as measured by insertion torque and ISQ values in animal bone blocks. However, these findings must be interpreted cautiously. The present investigation was conducted under controlled in vitro conditions and evaluated only immediate mechanical parameters. Therefore, the results cannot be directly extrapolated to biological processes such as osseointegration, long-term implant stability, or clinical success in humans. While certain drilling protocols showed higher primary stability under the tested conditions, well-designed prospective clinical studies are required to determine their true clinical relevance and predictability in patients.

Among the study’s strengths are the large sample size (722 implants), the standardized drilling process with calibrated stops, and the use of natural bone with controlled densities. This allowed for a robust evaluation of multiple experimental conditions.

Future multicenter clinical studies are needed to corroborate these results and establish safe torque limits for extra-short implants of different designs and surfaces.

## 5. Conclusions

Within the limitations of this ex vivo animal bone model study, the results indicate that different drilling protocols significantly influence the primary mechanical stability of ultra-short implants, as measured by insertion torque and ISQ values. Certain drilling protocols resulted in higher insertion torque and ISQ compared to others, particularly in Type II and Type III bone. However, these findings are restricted to immediate mechanical parameters under controlled in vitro conditions. Further clinical studies are necessary to evaluate the clinical relevance of these drilling protocols and their potential impact on implant treatment outcomes.

## Figures and Tables

**Table 1:** Implants included in the study and corresponding drilling protocols.

Preparation Type	Involved Lines	Affected Implants	Main Notes
Standard	L1, L3, L5, L7	Straumann SP 4.1 (4 & 5 mm), Klockner Ec 4.0/4.5 (4 mm)	Reference/manufacturer-recommended protocol
Horizontal underdrilling	L2, L4	Straumann SP 4.1 (4 & 5 mm)	Reduced diameter to 3.5 mm
Vertical underdrilling 1 mm	L6, L8	Klockner Ec 4.0/4.5 (5 mm)	Infragingival (extra depth)
Final width 3.5 mm	L9, L11	Klockner Ec 4.0/4.5 (4 mm)	Narrow/horizontal preparation
Combined (Vertical + 3.5 mm width)	L10, L12	Klockner Ec 4.0/4.5 (5 mm)	Maximum restriction (most aggressive)

**Table 2:** Kruskal–Wallis Test applied to the Torquemeter variable in B2 type III bone.

Drilling	*N*	Median	Ave Rank	Z
1 mm deeper	30	21.00	65.3	0.88
Narrower + deeper	30	25.00	72.9	2.25
narrower	30	19.00	56.4	−0.75
Fab recomende	30	15.00	47.4	−2.38
Overall	120		60.5	

H = 9.08 DF = 3 *P* = 0.028; H = 9.10 DF = 3 *P* = 0.0028 (adjusted for ties).

**Table 3:** Kruskal–Wallis Test related to the average ISQ in B2 type III bone.

Drilling	*N*	Median	Ave Rank	Z
1 mm deeper	30	59.50	65.6	0.93
Narrower + deeper	30	62.75	87.0	4.82
narrower	30	54.75	46.8	−2.49
Fab recomende	30	54.75	42.5	−3.26
Overall	120		60.5	

H = 30.769 *P* = 9.507 × 10^−7^; H = 30.77 DF = 3 *P* = 0.000 (adjusted for ties).

**Table 4:** Kruskal–Wallis Test applied to the torquemeter variable in B2 type II bone.

Drilling	*N*	Median	Ave Rank	Z
1 mm deeper	31	66.00	95.5	6.36
Narrower + deeper	30	31.00	40.01	−3.77
narrower	30	27.50	29.6	−5.66
Fab recommended	30	50.00	77.7	3.01
Overall	121		61.0	

H = 71.747 DF = 3 *P* = 1.804 × 10^−15^; H = 71.75. DF = 3 *P* = 0.000 (adjusted for ties).

**Table 5:** Kruskal–Wallis Test applied to the average ISQ variable in B2 type II bone.

Drilling	*N*	Median	Ave Rank	Z
1 mm deeper	31	66.50	75.5	2.67
Narrower + deeper	30	52.25	45.9	−2.72
narrower	30	54.00	44.5	−2.96
Fab recommended	30	66.50	77.5	2.98
Overall	121		61.0	

H = 24.189 DF = 3 *P* = 2.281 × 10^−15^; H = 24.19 DF = 3 *P* = 0.000 (adjusted for ties).

**Table 6:** Kruskal–Wallis Test applied to the average torquemeter variable in B1 type II bone.

Drilling	*N*	Median	Ave Rank	Z
1 mm deeper	30	60.00	72.8	2.24
Narrower + deeper	30	56.50	88.5	5.08
narrower	30	33.00	38.0	−4.08
Fab recommended	30	35.00	42.7	−3.24
Overall	120		60.5	

H = 43.557 DF = 3 *P* = 1.856 × 10^−9^.

**Table 7:** Kruskal–Wallis Test applied to the average ISQ variable in B1 type II bone.

Drilling	*N*	Median	Ave Rank	Z
1 mm deeper	30	67.00	71.5	1.99
Narrower + deeper	30	64.50	65.8	0.96
narrower	30	63.75	58.9	−0.29
Fab recomende	30	62.50	45.9	−2.66
Overall	120		60.5	

H = 9.04 DF = 3 *P* = 0.029; H = 9.05 DF = 3 *P* = 0.029 (adjusted for ties).

## Data Availability

The original contributions presented in this study are included in the article. Further inquiries can be directed to the corresponding author.

## Abbreviations

C/I	Crown-implant ratio
ISQ	Implant Stability Quotient
EC	Essential Cone
SP	Standard Plus
SLA	Sand Blasting Acid
RFA	Radio Frequency Analysis
IT	Insertion Torque
Ncm	Newton/cm
